# Biological Recognition-Based Electrochemical Aptasensor for Point-of-Care Detection of cTnI

**DOI:** 10.3390/bios13070746

**Published:** 2023-07-19

**Authors:** Jianfeng Ma, Lin Feng, Jie Li, Dan Zhu, Lianhui Wang, Shao Su

**Affiliations:** State Key Laboratory of Organic Electronics and Information Displays & Jiangsu Key Laboratory for Biosensors, Institute of Advanced Materials (IAM), Nanjing University of Posts and Telecommunications, 9 Wenyuan Road, Nanjing 210023, China; m18438606992@163.com (J.M.); kitten_fl@icloud.com (L.F.); iamdzhu@njupt.edu.cn (D.Z.)

**Keywords:** electrochemical detection, aptasensor, cardiac troponin I, point-of-care testing

## Abstract

As a “gold standard biomarker”, cardiac troponin I (cTnI) is widely used to diagnose acute myocardial infarction (AMI). For an early clinical diagnosis of AMI, it is necessary to develop a facile, fast and on-site device for cTnI detection. According to this demand, a point-of-care electrochemical aptasensor was developed for cTnI detection by coupling the advantages of screen-printed carbon electrode (SPCE) with those of an aptamer. Thiol and methylene blue (MB) co-labelled aptamer (MB-Apt-SH) was assembled on the surface of hierarchical flower-like gold nanostructure (HFGNs)-decorated SPCE (SPCE-HFGNs) to recognize and analyze cTnI. In the presence of cTnI, the specific biological recognition reaction between cTnI and aptamer caused the decrease in electrochemical signal. Under the optimal condition, this designed aptasensor showed wide linear range (10 pg/mL–100 ng/mL) and low detection limit for (8.46 pg/mL) for cTnI detection with high selectivity and stability. More importantly, we used a mobile phone coupled with a simple APP to efficiently detect cTnI in 10 μL 100% human serum samples, proving that this aptasensor has a promising potential in point-of-care testing.

## 1. Introduction

Acute myocardial infarction (AMI) is a serious cardiovascular disease that may lead to irreversible myocardial injury or necrosis [1,2]. Clinical data have showed that electrocardiogram (ECG) is not sufficient to accurately determine AMI due to an absence of significant ECG changes in nearly half of AMI patients [3,4,5]. Therefore, many AMI patients might miss the best treatment time, leading to prolongation of their illness and even endangering their lives. Accordingly, the development of a rapid, accurate, inexpensive and portable detection device for AMI early diagnosis can efficiently improve patients’ survival rates.

It is well-known that cardiac troponin I (cTnI) is a powerful biomarker for AMI diagnosis due to the direct relationship between its abnormal increasing concentration and myocardial contraction [6,7,8]. Accurate and fast detection of cTnI plays a vital role in the early diagnosis of AMI [9]. Among the different detection strategies, an electrochemical biosensor is a promising tool for the detection of cTnI due to its simple, fast, accurate and easy device application [10,11,12,13]. For example, Cheng et al. developed an electrochemical immunosensor for the highly sensitive detection of cTnI by using the ring-opening polymerization (ROP) reaction as a signal amplification strategy [14]. Zhang’s group designed an electrochemical immunosensor for cTnI detection by utilizing covalent organic frameworks as signal amplification probes and toluidine blue as signal molecules, which can detect cTnI as low as 0.17 pg/mL without introducing additional signal indicators [15]. The great advances of electrochemical biosensors offered a possibility to sensitively and selectively determine cTnl for the early diagnosis of AMI.

Compared with antibody, aptamer is considered an ideal recognition unit for the detection of cTnI due to its advantages of high binding affinity, excellent specificity, good stability and easy modification [16,17,18]. Based on this concept, many electrochemical aptasensors have been designed for the detection of cTnI coupled with signal amplification strategies or modification methods of electrodes [11,19,20,21,22]. For example, Sun’s group used natural enzyme and hybridized nanozyme as co-catalysis to construct a signal-amplified electrochemical aptasensor. As expectedly, this aptasensor can quantitatively detect 0.01–100 ng/mL cTnI with a detection limit of 7.5 pg/mL [19]. Jo’s group coupled the advantages of screen-printed carbon electrode (SPCE) with an aptamer to construct a sensitive electrochemical aptasensor for the detection of cTnI [23]. The experimental results suggested that this portable SPCE-based aptasensor will become an innovative diagnostic tool for AMI diagnosis.

**Scheme 1:**
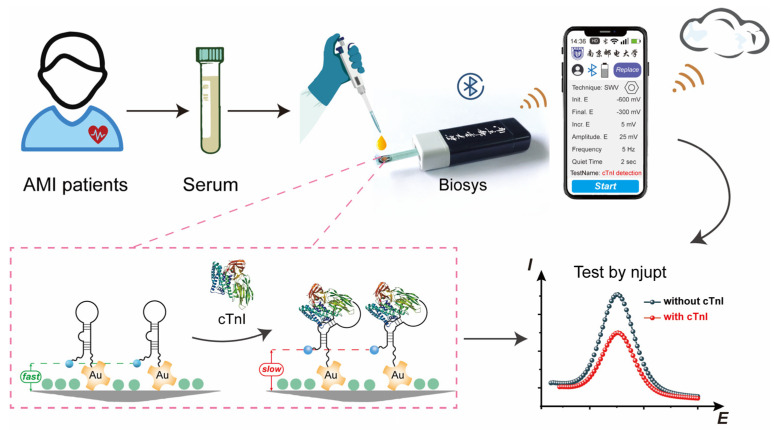
Schematic illustration of an electrochemical aptasensor for the point-of-care detection of cTnI based on biological recognition.

For point-of-care testing (POCT) of cTnI, a crucial problem is to construct simple, efficient and stable detection system and device [24,25]. In this study, an efficient and convenient electrochemical aptasensor was developed for POCT of cTnI based on SPCE. To efficiently improve electrochemical performance, three-dimensional hierarchical flower-like gold nanostructures (HFGNs) were electrodeposited on the surface of SPCE. As shown in Scheme 1, the target-recognizing aptamer (Apt) labelled with methylene blue (MB) is assembled onto the electrode surface via classical Au-S bond. Expectedly, Apt specifically recognized with cTnI and formed an Apt-cTnI complex, which greatly hinders the electron transfer between MB and the electrode surface. As a result, the electrochemical signal decreased with the addition of cTnI. According to this phenomenon, this aptasensor can qualitatively and quantitatively detect cTnI with excellent performance. Coupled with portable electrochemical device and phone APP, the designed aptasensor can also efficiently analyze cTnI. It should be pointed out that the developed electrochemical device can accurately determine cTnI in 10 µL 100% human serum sample within 10 min. Thus, the proposed device is suitable for rapid and on-site detection of cTnI, which has promising application potential in ambulances or emergency situations for the early diagnosis of AMI.

## 2. Materials and Methods

### 2.1. Materials and Reagents

The aptamer was synthesized by Sangon Biotech Co., Ltd. (Shanghai, China), which was listed in Table S1. SPCE was purchased from Ningbo Yuangan Biotechnology Co., Ltd. (Ningbo, China). Cardiac troponin I (cTnI), cardiac troponin C (cTnC), cardiac troponin T (cTnT), N-terminal forebrain natriuretic peptide (BNP) and human serum albumin (HSA) were purchased from Shanghai Linc-Bio Science Co., Ltd. (Shanghai, China). Healthy human serum was purchased from Shanghai Jiwei Biotechnology Co., Ltd. (Shanghai, China). Chloroauric acid trihydrate (HAuCl_4_·3H_2_O, ≥99%), polyacrylic acid (PAA), polyethyleneimine (PEI), tris(2-carboxyethyl) phosphine (TCEP) and 6-mercapto-1-hexanol (MCH) were obtained from Sigma-Aldrich Co., Ltd. (Shanghai, China). Disodium hydrogen phosphate dodecahydrate (Na_2_HPO_4_·12H_2_O, ≥99.0%), sodium dihydrogen phosphate dihydrate (NaH_2_PO_4_·2H_2_O, ≥99.0%), magnesium chloride (MgCl_2_), sodium chloride (NaCl) and potassium chloride (KCl) were purchased from Sinopharm Chemical Reagent Co., Ltd. (Shanghai, China). Phosphate buffer (PB) and phosphate-buffered saline (PBS) were prepared using NaH_2_PO_4_ (0.2 M), Na_2_HPO_4_ (0.2 M) and different salts according to the standard protocol. All reagents were of analytical grade and used without further purification. Aqueous solutions were prepared by using ultrapure water (>18 MΩ·cm) obtained from the Millipore water purification system.

### 2.2. Apparatus

The morphology of SPCE-HFGNs was characterized using scanning electron microscope (SEM, S-4800, HITACHI, Tokyo, Japan). Cyclic voltammetry (CV) and square wave voltammetry (SWV) measurements were performed on a CHI660D electrochemical workstation (Shanghai, China) and intelligent electrochemical workstation (BioSYS, Shanghai, China). The working electrode, reference electrode and auxiliary electrode of a typical SPCE were carbon, Ag/AgCl and carbon, respectively.

### 2.3. Fabrication and Evaluation of Electrochemical Aptasensor

SPCE-HFGNs were prepared according to the procedures described in our previous work [26,27]. After preparation, thiol and methylene blue (MB) co-labelled aptamer (MB-Apt-SH) was assembled on the surface of SPCE-HFGNs via Au-S bond for 12 h, producing a modified electrode (named as MB-Apt-SH/SPCE-HFGNs). Then, MCH was used to block non-specific binding sites of MB-Apt-SH/SPCE-HFGNs. After drying, 10 μL of different concentrations of cTnI was incubated onto the electrode surface for 10 min at room temperature. Finally, the MB-Apt-SH/SPCE-HFGNs was tested via different electrochemical methods for qualitative and quantitative detection of cTnI. The scanning potential window of square wave voltammetry (SWV) ranged from −0.3 to −0.6 V with an amplitude of 25 mV and a frequency of 50 Hz in PBS (0.01 M PB, 150 mM NaCl, pH 7.4). The as-prepared electrodes were stored at 4 °C in refrigerator to evaluate the stability of this aptasensor. To obtain the best performance of the electrochemical aptasensor, the experimental conditions were optimized, including the concentration of the aptamer, the MCH concentration and the detection time (Figure S1 in Supplementary Materials).

### 2.4. Construction of POCT Electrochemical System for cTnI Detection

The handheld electrochemical system was tested by refreshing the intelligent electrochemical workstation (BioSYS). First, the as-prepared MB-Apt-SH/SPCE-HFGNs was inserted into the USB-shaped electrochemical station. Second, a phone app was connected with the USB-shaped electrochemical station via Bluetooth technology. Third, 10 μL 100% healthy human serum containing different concentrations of cTnI was dropped on the surface of MB-Apt-SH/SPCE-HFGNs for incubation 10 min. Finally, the electrochemical signal was recorded in the phone app. It should be pointed out that the detection results were uploaded to the cloud through website timely, which can be viewed everywhere at any time and saved permanently.

## 3. Results

### 3.1. Fabrication and Characterization of SPCE-HFGNs

**Figure 1:**
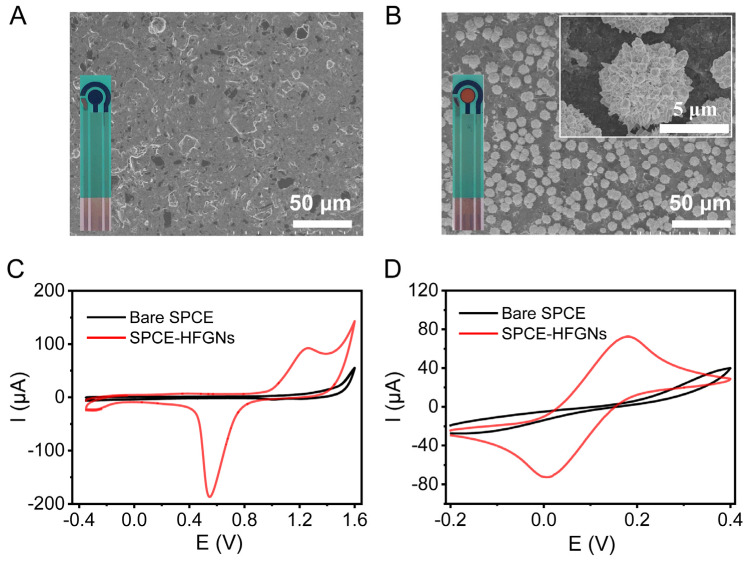
SEM images of (**A**) SPCE and (**B**) SPCE-HFGNs. Scale bars: 50 µm. Insets: corresponding photos of SPCE and SPCE-HFGNs. (**C**) CVs of SPCE and SPCE-HFGNs in H_2_SO_4_ solution. (**D**) CVs of SPCE and SPCE-HFGNs in K_3_[Fe (CN)_6_]/K_4_[Fe (CN)_6_] solution containing 0.1 M KCl.

As shown in Figure 1A, bare SPCE showed typical nano-roughened carbon porous structures. After electrodeposition, well-defined and uniform HFGNs with a diameter of 5 µm were deposited on the surface of SPCE (Figure 1B). In addition, an obvious golden color was obtained on SPCE-HFGNs (inset in Figure 1B), indicating the successful deposition of gold nanostructures. The electrochemical behavior of SPCE-HFGNs was characterized via cyclic voltammetry (CV) in 0.5 M H_2_SO_4_. As shown in Figure 1C, no redox peaks were observed at SPCE (black curve). After the modification of HFGNs, significant gold redox peaks were obtained at SPCE-HFGNs (red curve), proving that HFGNs electrodeposited on the surface of SPCE. Moreover, the electrochemical behaviors of bare SPCE and SPCE-HFGNs were also studied in the [Fe(CN)_6_]^3−/4−^ solution. Obviously, the electrochemical performance of SPCE-HFGNs was greatly improved after the decoration of HFGNs (Figure 1D). A pair of well-defined redox peaks were observed at SPCE-HFGNs. The electrochemical currents of SPCE-HFGNs were almost 1.7-fold that of SPCE. All experimental data indicate that the electrodeposition of HFGNs can efficiently improve the electrochemical performance of SPCE, which is a promising electrode for the construction of electrochemical sensors.

### 3.2. Feasibility of the Electrochemical Aptasensor

**Figure 2:**
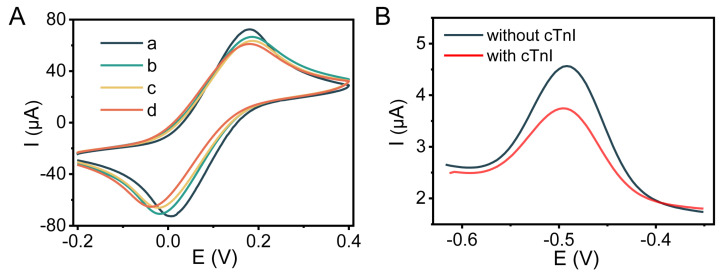
(**A**) CV curves of different electrodes (a) SPCE-HFGNs, (b) MB-Apt-SH/SPCE-HFGNs, (c) MCH/MB-Apt-SH/SPCE-HFGNs and (d) cTnI/MCH/MB-Apt-SH/SPCE-HFGNs in 5 mM K_3_[Fe (CN)_6_]/K_4_[Fe (CN)_6_] solution containing 0.1 M KCl. (**B**) SWV responses of the designed aptasensor with and without cTnI in PBS (0.01 M PB, 150 mM NaCl, pH 7.4).

The preparation process of this aptasensor was characterized. As shown in Figure 2A, a pair of reversible oxidation and reduction peaks of the electrochemical indicator ([Fe (CN)_6_]^3−/4−^) were obtained at SPCE-HFGNs, which is attributed to the excellent electrochemical properties of SPCE-HFGNs (curve a). After incubation with Apt, the redox peak currents decreased, indicating that Apt was successfully immobilized on the surface of SPCE-HFGNs (curve b). The reason is ascribed to the negatively charged Apt hindering the electron transfer between [Fe (CN)_6_]^3−/4−^ and the electrode surface [28,29]. With the blocking of MCH, the reduction and oxidation peaks currents further reduced, indicating that MCH efficiently blocks the nonspecific binding sites of SPCE-HFGNs (curve c). After incubating 50 ng/mL cTnI, Apt formed a secondary or tertiary structure to specifically bind with cTnI. As a result, the peak currents further decreased due to the biological recognition of Apt towards cTnI (curve d). The above results proved the successful construction of the electrochemical aptasensor.

The feasibility of this aptasensor for the detection of cTnI was studied via square wave voltammetry (SWV). As shown in Figure 2B, an obvious oxidation peak was observed at MB-Apt-SH/SPCE-HFGNs, which was ascribed to the labelled MB indicator (black curve). Obviously, a biological recognition reaction occurred with the addition of 50 ng/mL cTnI due to the specific binding ability of Apt towards cTnI. The formation of the cTnI-Apt complex inhibited the electron transfer between MB and electrode surface, leading to the decrease in the electrochemical response of MB-Apt-SH/SPCE-HFGNs (red curve). This experimental result showed that the designed electrochemical aptasensor can efficiently recognize and analyze cTnI.

### 3.3. Detection Performance of the Electrochemical Aptasensor

**Figure 3:**
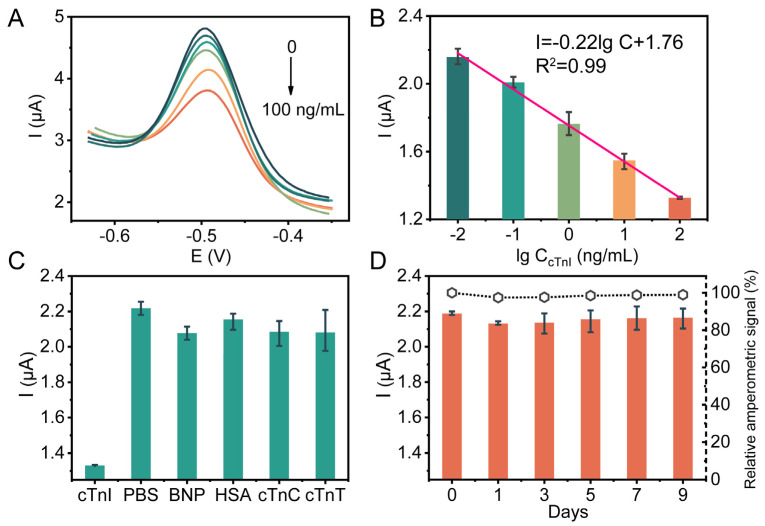
(**A**) SWV responses of the aptasensor for the detection of different cTnI concentrations in PBS (0.01 M PB, 150 mM NaCl, pH 7.4) (from top to bottom): 0 pg/mL, 10 pg/mL, 100 pg/mL, 1 ng/mL, 10 ng/mL and 100 ng/mL, respectively. (**B**) Calibration plots between the SWV current and cTnI concentration ranging from 10 pg/mL to 100 ng/mL. (**C**) The corresponding current of this biosensor for the detection of PBS, BNP, HSA, cTnC, cTnT and cTnI. (**D**) The storage stability of this aptasensor. The error bars represent the standard deviation of at least three independent measurements.

Before detection, the effect of experimental conditions on the analytical performance of this aptasensor were tested (Figure S1). Under optimal conditions, the analytical performance of this aptasensor was investigated. Figure 3A shows the SWV responses of the aptasensor for different concentrations of cTnI detection. Obviously, the peak currents of this aptasensor decreased with the addition of 10 pg/mL–100 ng/mL cTnI. A linear range between peak currents and the logarithm concentration of cTnI was obtained with an equation of I = −0.22l g C_cTnI_ + 1.76 (Figure 3B). The limit of detection (LOD) of this aptasensor was estimated to be 8.46 pg/mL, which is lower than the clinical diagnostic concentration [30] (about 400 pg/mL). Moreover, the detection performance of this aptasensor is comparable to or better than that of some published works, suggesting that our developed electrochemical aptasensor has promising potential in AMI early diagnosis (Table 1).

As we know, B-type natriuretic peptide (BNP), cardiac troponin C (cTnC), cardiac troponin T (cTnT), and cardiac troponin I (cTnI) are potential biomarkers for the rapid diagnosis of AMI [9,31,32]. Therefore, the selectivity of this aptasensor was evaluated by using PBS, HSA, BNP, cTnC and cTnT as interfering substances. As shown in Figure 3C, 100 ng/mL cTnI could trigger a significant decrease in the electrochemical signal, while 10-fold HSA, BNP, cTnC or cTnT only caused a small change (˂30%) in SWV signals, indicating that this aptasensor has an accepted selectivity. Subsequently, six batches of MB-Apt-SH/SPCE-HFGNs were selected to test the reproducibility of this aptasensor. For 5 ng/mL cTnI detection, the relative standard deviation (RSD) of this aptasensor was only 1.93%, demonstrating that this aptasensor has excellent reproducibility (Figure S2). Moreover, the stability of this electrochemical aptasensor was also evaluated (Figure 3D). After 9-day storage, the peak current of this aptasensor was maintained at about 95% of its original current, suggesting that this aptasensor has excellent stability.

**Table 1:** Comparison of the analytical performance of the developed electrochemical aptasensor with other reported biosensors for cTnI detection.

Electrode	Dynamic Range (ng/mL)	LOD (pg/mL)	Ref.
P2-SH/Au	0.5–100	40	[10]
DNA nanotetrahedron-Apt/SPGE	0.05–100	16	[33]
Ab/WNFs/GCE	0.5–2	40	[34]
Apt-SH/ND-Au	0.05–500	8	[35]
Apt-SH/SPCE-AuNPs	0.024–2.4	24	[23]
Ab1/Au	0.005–10	1.7	[36]
MB-Apt-SH/SPCE-HFGNs	0.01–100	8.46	This work

[SPGE] screen-printed gold electrode, [WNFs] whiskered nanofibers, [ND-Au] gold nanodumbbells, [Ab] antibody.

### 3.4. Application of the Electrochemical Aptasensor in Serum Samples

**Figure 4:**
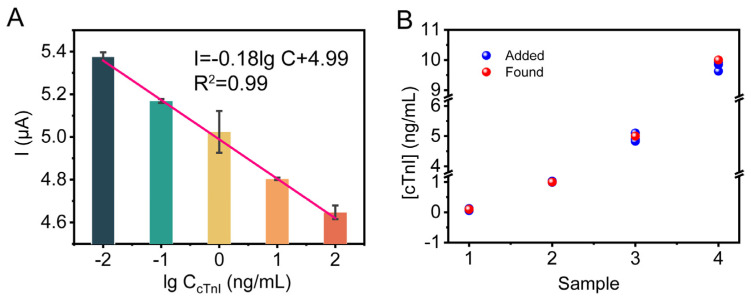
(**A**) The relationship between the SWV current and the logarithm concentration of cTnI ranging from 10 pg/mL–100 ng/mL in healthy human serum. (**B**) The aptasensor for the detection of 100 pg/mL, 1 ng/mL, 5 ng/mL and 10 ng/mL cTnI in human serum, respectively. The error bars represent the standard deviation of at least three independent measurements.

In clinical studies, the concentration of cTnI in serum ranging from 0.5 to 2.0 ng/mL is considered the boundary between healthy individuals and patients [9,31,32,36]. To verify the clinical application of this biological recognition-based electrochemical aptasensor, MB-Apt-SH/SPCE-HFGNs was applied to determine cTnI in healthy human serum (Figure 4A). The electrochemical signal decreased continuously with the increasing concentration of cTnI in the sample. A linear relationship between the peak current and the logarithm concentration of cTnI (10 pg/mL–100 ng/mL) was obtained using the following equation: I = −0.18l g C_cTnI_ + 4.99 (Figure 4A). According to the equation, the practicability of the proposed aptasensor was investigated. As shown in Figure 4B, the recovery and relative standard deviation (RSD) of the proposed aptasensor for the detection of cTnI was about 96.33–101.15% and 1.52–3.29% (for detailed data, please see Table S2), respectively, suggesting that the constructed aptasensor has potential for clinical applications.

### 3.5. Fully Integrated Handheld cTnI-Sensing Device

**Figure 5:**
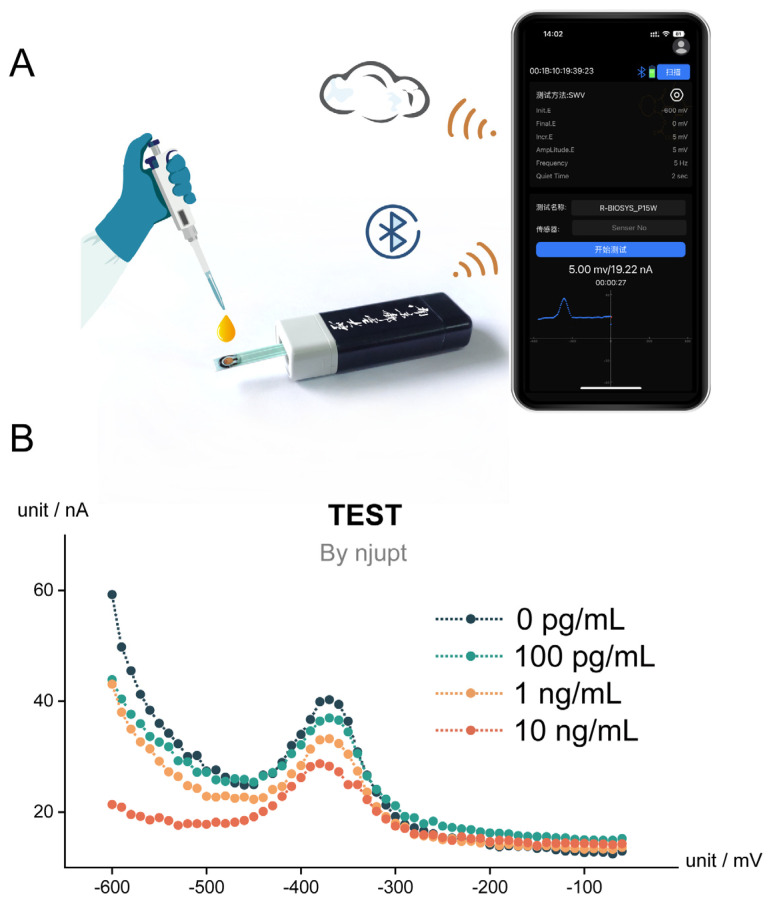
(**A**) Schematic illustration of the handheld cTnI–sensing device. (**B**) SWV responses of the aptasensor for the detection of different cTnI concentrations in healthy human serum by using the POCT device.

According to above exciting results, a fully integrated POCT electrochemical system was designed by integrating MB-Apt-SH/SPCE-HFGNs with a BioSYS Toolkit (Figure 5A). Notably, the electrochemical data were not only directly displayed on the phone, but could also be saved in the cloud via the website. In other words, these data can be viewed anywhere at any time. Figure 5B depicts the performance of the fully integrated cTnI-sensing device for 100 pg/mL, 1 ng/mL, and 10 ng/mL cTnI detection, respectively. The experimental results show that the fully integrated cTnI-sensing device can qualitatively and quantitatively detect cTnI in 10 μL 100% human serum without further washing, suggesting that the proposed device has promising applications in the early diagnosis of AMI.

## 4. Conclusions

In summary, we developed a biological recognition-based electrochemical aptasensor for point-of-care cTnI detection. Benefiting from the biological recognition ability of aptamer and excellent electrical conductivity of SPCE-HFGNs, this aptasensor exhibits a low detection limit of 8.46 pg/mL and a wide dynamic range of 10 pg/mL–100 ng/mL. More importantly, we integrated the aptasensor with a handheld electrochemical system and a custom-developed mobile application for point-of-care monitoring of cTnI. This cTnI-sensing device paves the way for the development of household devices for the early diagnosis of AMI.

## Data Availability

Not applicable.

## Supplementary Materials

The following supporting information can be downloaded at: https://www.mdpi.com/article/10.3390/bios13070746/s1, Figure S1: Optimization of experimental conditions; Figure S2: Reproducibility of this biosensor; Table S1: All sequences used in this experiment; Table S2: The recovery and RSD of this biosensor for cTnI detection in serum samples.
