# Supplementary material for: Biological Recognition-Based Electrochemical Aptasensor for Point-of-Care Detection of cTnI
Source: Biosensors (Basel). 2023 Jul 19;13(7):746. doi: 10.3390/bios13070746 (PMC10377036; doi:10.3390/bios13070746)
Supplement: Supplementary file 1 [file biosensors-13-00746-s001.zip › biosensors-2490996-supplementary.pdf]

SI APPENDIX

Biological Recognition-Based Electrochemical Aptasensor for Point-of-Care Detection of cTnI

Jianfeng Ma[1], Lin Feng[1], Jie Li[1], Dan Zhu[1], Lianhui Wang[1,]* and Shao Su[1,]*

[1] State Key Laboratory of Organic Electronics and Information Displays & Jiangsu Key Laboratory for Biosensors, Institute of Advanced Materials (IAM), Nanjing University of Posts and Telecommunications, 9 Wenyuan Road, Nanjing 210023, China
* Correspondence: iamlhwang@njupt.edu.cn (L.W.); iamssu@njupt.edu.cn (S.S.)

In order to obtain the best performance of the electrochemical aptasensor, the experimental conditions were optimized, including the concentration of the aptamer, the MCH concentration and the detection time. First, the concentration of Apt was optimized. The result showed that the optimized concentration was 0.5 μM (Figure S1A). The concentration of MCH was also optimized in Figure S1B. The optimal concentration was 1 mM MCH. As shown in Figure S1C, 20 min was the optimal detection time. However, as the sensor already has good detection performance at 10 minutes, 10 minutes was chosen as the final detection time of the aptasensor.

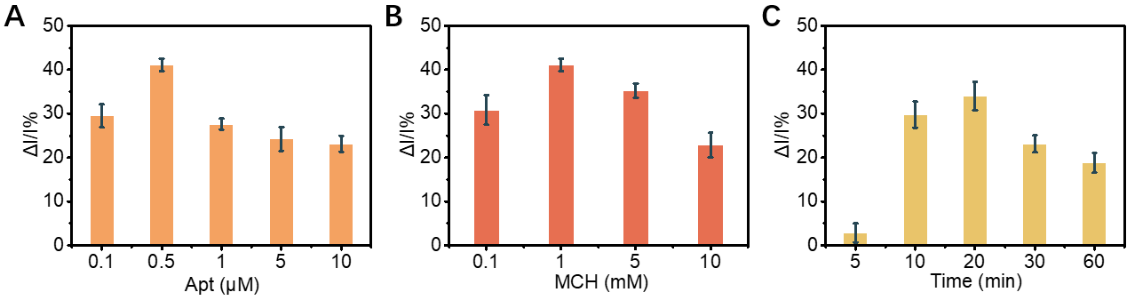

Figure S1. Optimization of the experimental conditions: (A) Apt concentration, (B) MCH concentration and (C) detection time, where the current signals of the sensor before and after incubation with cTnI are I and I_{cTnI}, respectively, and the signal change rate $\Delta I/I\% = (I - I_{cTnI})/I \times 100\%$.

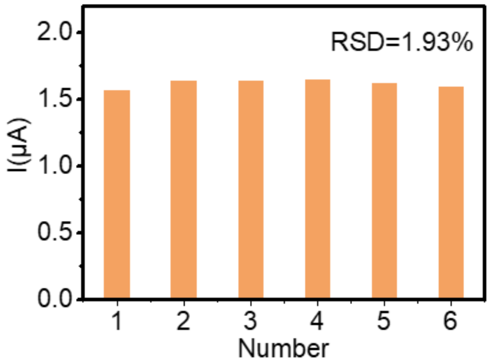

Figure S2. Reproducibility of this biosensor for cTnI detection by using six independently electrodes.

Table S1. All sequences used in this experiment.

Name	Sequence (5’-3’)
Apt	SH-CGTGCAGTACGCCAACCTTTCTCATGCGCTGCCCCTCTTA-MB

Table S2. The recoveries of serum samples were detected by this biosensor.

Sample	Added (ng/mL)	Detected (ng/mL)	RSD %	Recovery %
1	0.1	0.109, 0.121, 0.095	3.29%	96.33%
2	1	0.99, 1.03, 1.01	2.30%	97.97%
3	5	4.89, 4.83, 5.10	2.87%	98.80%
4	10	9.63, 9.85, 9.92	1.52%	101.15%
